# Leaf-Encapsulated Vaccines: Agroinfiltration and Transient Expression of the Antigen *Staphylococcal Endotoxin* B in Radish Leaves

**DOI:** 10.1155/2018/3710961

**Published:** 2018-02-07

**Authors:** Pei-Feng Liu, Yanhan Wang, Robert G. Ulrich, Christopher W. Simmons, Jean S. VanderGheynst, Richard L. Gallo, Chun-Ming Huang

**Affiliations:** ^1^Department of Dermatology, University of California, San Diego, CA 92161, USA; ^2^Department of Immunology, Army Medical Research Institute of Infectious Diseases, Frederick, MD 21703, USA; ^3^Department of Biological & Agricultural Engineering, University of California, Davis, CA 95616, USA; ^4^Department of Dermatology and Moores Cancer Center, University of California, San Diego, CA 92161, USA

## Abstract

Transgene introgression is a major concern associated with transgenic plant-based vaccines. Agroinfiltration can be used to selectively transform nonreproductive organs and avoid introgression. Here, we introduce a new vaccine modality in which Staphylococcal enterotoxin B (SEB) genes are agroinfiltrated into radishes (*Raphanw sativus* L.), resulting in transient expression and accumulation of SEB *in planta*. This approach can simultaneously express multiple antigens in a single leaf. Furthermore, the potential of high-throughput vaccine production was demonstrated by simultaneously agroinfiltrating multiple radish leaves using a multichannel pipette. The expression of SEB was detectable in two leaf cell types (epidermal and guard cells) in agroinfiltrated leaves. ICR mice intranasally immunized with homogenized leaves agroinfiltrated with SEB elicited detectable antibody to SEB and displayed protection against SEB-induced interferon-gamma (IFN-*γ*) production. The concept of encapsulating antigens in leaves rather than purifying them for immunization may facilitate rapid vaccine production during an epidemic disease.

## 1. Introduction

Transgenic plants have emerged as a promising technology to generate recombinant biopharmaceutical proteins and vaccines [1, 2]. Plants produce full-length mammalian proteins that appear to be processed correspondingly to their native counterpart with appropriate folding, assembly, and posttranslational modifications [3]. Although stably transformed transgenic plants have been widely created to deliver edible vaccines [4, 5] and have proven success in clinical trials [6, 7], the fact that transgenes are permanently incorporated into the genomes of transgenic plants raises many concerns, such as the environmental release of genetically modified plants and the possibility of transgene introgression into nonmodified counterparts [8]. In addition, immunization with edible vaccines derived from transgenic plants may carry a risk of inducing oral tolerance due to immunization with multidoses within a long period of time. Transient expression of recombinant proteins in leaf tissue avoids transgene introgression and provides a fast platform for protein production without an effort-exhaustive process to generate stably transformed transgenic plants [9].

Currently, there are at least four approaches to transforming and inducing transient expression in plants: (1) delivery of “naked” DNA by particle bombardment [10], (2) infection with modified viral vectors [6, 10, 11], (3) agroinfiltration of plant tissues with *Agrobacteria* [10, 12], and (4) polyethylene glycol- (PEG-) mediated gene transfer and electroporation of protoplasts [13]. Agroinfiltration accommodates transforming plants with large genes encoding complex proteins, such as antibodies. Moreover, agroinfiltration-induced transient expression can yield high levels of recombinant protein [14]. Vacuum and syringe infiltration are two major methods of promoting agroinfiltration and expressing proteins/antigens in plants [15, 16]. Unlike the vacuum infiltration, syringe infiltration can be easily applied for infiltrating multiple antigens on the same leaf. Syringe infiltration, where a needle-less syringe is placed at the surface of a leaf and used to push a suspension of *A. tumefaciens* into the leaf interior, provides a high level of control over which tissues are transformed. In contrast to agroinfiltration, the efficiency of particle bombardment using a gene gun is relatively low since transgenes are successfully delivered to only few target cells [14]. Furthermore, transient expression using plant virus infection shows many disadvantages, such as biosafety and construct-size limitation [2]. Protoplast transformation involves a care-intensive, complicated procedure of isolating protoplasts from leaf mesophylls. Protoplasts can also respond differently from intact cells and may not be suitable for certain types of expression analysis [17].

Staphylococcal enterotoxin B (SEB) is one of the several toxins produced by *Staphylococcus aureus* bacteria [18, 19]. The toxin commonly causes outbreaks of food poisoning. Also, SEB has been studied as a potential biological warfare agent because it can easily be aerosolized, is very stable, and can cause shortness of breath, widespread systemic damage, and even shock and death when inhaled at very high dosages [20–22]. Molecularly, SEB acts as a superantigen, binding to class II major histocompatibility complex proteins and stimulating T cells to induce inflammation and cytokine (e.g., tumor necrosis factor alpha and interferon-gamma (IFN-*γ*)) release [23]. Considering the toxicity and potential weaponization of SEB, there is an urgent need to have anti-SEB vaccines that can be produced in an effortless and timely manner during SEB outbreaks.

Here, we generate SEB vaccines by agroinfiltrating SEB genes into radish leaves. Intranasal immunization of mice with SEB-expressing leaves in conjunction with adjuvant cholera toxin (CT) elicited systemic antibodies to SEB and offered protective immunity against SEB-induced IFN-*γ* production. We also demonstrate that two different antigens (SEB and a tetanus toxin C fragment (TetC)) can be simultaneously agroinfiltrated and transiently expressed within the same leaf. Notably, we here highlight the concept of stamping antigens onto leaves to generate vaccines by using agroinfiltration. The technique shows that agroinfiltration can be used to rapidly induce transient expression of antigens in leaf tissue, which can be used for immunization in a way that eliminates complicated purification procedures commonly associated with recombinant antigens. This work illustrates that agroinfiltrated/stamped leaves can not only act as bioreactors for antigen production but may also serve as capsulated vaccines containing one or more antigens for patient immunization.

## 2. Materials and Methods

### 2.1. Plant Materials

Japanese radish sprouts (Kaiware-daikon) (*Raphanus sativus* L.) and lettuce (*Lactuca sativa*) were obtained from a commercial supplier (ICREST International, JCP, Carson, CA). Japanese radish sprouts that were 9 cm in length with two leaflets were used. *Arabidopsis thaliana* seeds were kindly provided by Professor Nigel Crawford at University of California, San Diego. All plants were grown at room temperature under a 23-watt fluorescent bulb (Philips, Portland, OR) and were sprayed with water daily.

### 2.2. Vector Construction and *Agrobacterium tumefaciens* Transformation

The methods of vector construction and transformation were according to a modified protocol described in our previous publication. Briefly, the binary vector pBI121 carrying the reporter GUS driven by the CaMV 35S promoter was used [24, 25]. A forward primer (5′-GATTCTAGAATGGAGAGTCAACCAGATCCTAAACCAGA-3′) and a reverse primer (5′-TCGCCCGGGCGCTTTTTCTTTGTCGTAAGATAAACTTC-3′) were utilized for polymerase chain reaction (PCR) to amplify the open reading frame of detoxified SEB cDNA with three mutations (National Center for Biotechnology Information (NCBI) accession number M11118) [26]. A forward primer (5′-GGATCTAGAATGGAAAATCTGGATTGTTGGG-3′) and a reverse primer (5′-AATCCCGGGCGGTCGTTGGTCCAACCTTC-3′) were added into a PCR reaction to amplify the TetC cDNA (NCBI accession number AM412776). PCR products were cloned into polylinker sites of pBI121 vectors to generate 35S::*SEB-GUS* and 35S::*TetC-GUS* constructs [25]. These two constructs were then transformed into *Agrobacterium tumefaciens* strain LBA4404 according to a liquid nitrogen freeze-thaw method.

### 2.3. Agroinfiltration of 35S::SEB-GUS and 35S::TetC-GUS Constructs into Radish Leaves

A single colony of *A. tumefaciens* transformants was cultured in 2 ml of YEP media (10 mg/ml Bacto™ Tryptone (DIFCO, Detroit, MI), 10 mg/ml yeast extract (DIFCO, Detroit, MI), and 5 mg/ml NaCl (Sigma, St. Louis, MO; pH 7.5)) containing 50 *μ*g/ml kanamycin and streptomycin at 28°C until optical density (OD) at 600 nm (OD_600_) reached 0.5. Nontransformed *Agrobacterium* served as a negative control. For syringe infiltration, as previously described [25], 0.1 ml of *Agrobacterium* bacterial suspension (5 × 10^7^ CFU) was injected into the wounded lower epidermis site for five days. For high-throughput agroinfiltration, six radish leaves were concurrently infiltrated with 0.1 ml of bacterial suspension containing the 35S::*SEB*-*GUS* construct using a multichannel pipette with open (2.2 mm diameter) tips. The infiltrated leaves were next placed in a dish containing wet cloths and incubated overnight.

### 2.4. Histochemical GUS Assays

Agroinfiltrated leaves were stained using a histochemical GUS assay solution consisting of 0.1 M NaPO_4_ (pH 7.0), 0.5 mM K_3_Fe(CN)_6_, 0.5 mM K_4_Fe(CN)_6_, 0.1% (*v*/*v*) Triton X-100, and 0.05% (*w*/*v*) X-Gluc (Sigma, St. Louis, MO) [27]. Leaves were submerged in the staining solution and incubated at 37°C in the dark overnight. After incubation, leaves were removed from the staining solution and immersed in a stop solution containing 42.5% (*v*/*v*) ethanol, 10% (*v*/*v*) formaldehyde, and 5% (*v*/*v*) acetic acid [28]. Stained leaves were embedded in OCT compound (Miles Inc., Diagnostics Division, Elkhart, IN) and cut with a glass knife on a cryogenic ultramicrotome (7 *μ*m thick). Fresh-mounted OCT sections were examined under bright-field microscopy (Olympus America, Inc., Melville, NY).

### 2.5. Intranasal Immunization with Homogenized Leaves Containing Recombinant SEB

Our previous study indicated that intranasal immunization of mice with ground leaves expressing CAMP factor elicits detectable antibodies to *P. acnes* CAMP factor, indicating that intranasal administration of whole plant leaves may be a new regimen for vaccination [25]. In the study, female ICR (Institute of Cancer Research) mice (3 to 6 weeks old; Harlan, Indianapolis, IN) were utilized for intranasal immunization. Intranasal immunization holds the potential to induce a mucosal immune response that recapitulates the natural SEB infection across the respiratory tract [29]. All mice used in the study were maintained in accordance to institutional IACUC guidelines. The central areas (25 mm^2^) of five radish leaves expressing SEB-GUS or GUS alone were excised using a sterile scalpel. Leaf sections were then pooled and homogenized under liquid nitrogen followed by addition of 700 *μ*l ddH_2_O and then sterilized by an ultraviolet crosslinker (Spectronics, Westbury, NY) at 7000 J/m^2^ for 30 min. Inactivation of sterilized *Agrobacterium* was confirmed by their inability to form colonies on YEP agar plates (data not shown). Twenty-five microliter homogenized leaves containing either SEB-GUS or GUS alone (as a negative control) mixed with a CT adjuvant (Sigma-Aldrich, St. Louis, MO) which has been used to boost the mucosal immunogenicity (5 *μ*g/25 *μ*l of ground leaf materials as described below) were then intranasally inoculated into the nasal cavities of ICR mice (25 *μ*l of ground leaf materials). Three boosts at the same dose were performed at 1, 2, and 4 weeks after the first immunization [30].

### 2.6. Western Blotting

Twenty *μ*g of homogenized leaves expressing either SEB-GUS or GUS alone were loaded into a 10% SDS-PAGE for antigen detection. After electrophoretically transferring SDS-PAGE to nitrocellulose membranes, the membranes were incubated with mouse monoclonal anti-SEB antibody (1 : 1000 dilution) (Toxin Technology, Sarasota, FL). To detect the production of antibodies in immunized mice, recombinant SEB (15 *μ*g) (Toxin Technology, Sarasota, FL) was subject to a 10% SDS-PAGE and transferred to a nitrocellulose membrane which was subsequently immunoreacted to four-week serum (1 : 500 dilution) obtained from mice immunized with whole leaf containing SEB-GUS. Immunoglobulin G (IgG) antibodies were detected with anti-mouse horseradish peroxidase-conjugated IgG (1 : 5000 dilution, Promega, Madison, WI). A Western Lighting™ Chemiluminescence kit (PerkinElmer, Boston, MA) was used to visualize the peroxidase activity.

### 2.7. Titration of Antibodies

The antibody titer of SEB was quantified by ELISA. Eight mice were used per group. Sera were collected 4 weeks after first immunization with L-GUS or L-SEB-GUS. Purified recombinant SEB (0.1 *μ*g/well) was diluted with PBS buffer and coated onto a 96-well ELISA plate (Corning, Lowell, MA) at 4°C overnight. The plate was washed with PBS containing 0.05% (*w*/*v*) Tween-20 and blocked with PBS containing 1% (*w*/*v*) bovine-serum albumin and 0.05% (*w*/*v*) Tween-20 for 2 h at room temperature. Pooled antisera obtained from eight immunized mice with L-GUS or L-SEB-GUS were serially diluted by 10-fold and separately added to the wells and incubated for 2 h. A goat anti-mouse IgG-HRP conjugate (Promega, Madison, WI) (1 : 5000 dilution) was added and incubated for 2 h before washing. HRP activity was determined with an OptEIA™ Reagent Set (BD Biosciences). The OD of each well was measured at 490 nm. The endpoint was defined as the dilution of sera producing the same OD at 490 nm as a 1/100 dilution of preimmune sera. Sera negative at the lowest dilution tested were assigned endpoint titers of 100. The data was presented as geometric mean endpoint ELISA titers as previously described [31].

### 2.8. Measurement of SEB-Induced IFN-*γ* Production in Immunized Mice

Naïve mice and immunized mice after the third boost were challenged intranasally with recombinant SEB (40 *μ*g/mouse) for overnight. Eight mice were used per group. After trachea cannulation, the lungs were lavaged twice with 0.5 ml of phosphate-buffered saline, and BAL fluids were pooled. After centrifugation at 1300g, IFN-*γ* in fluids pooled from eight mice per group was measured by an ELISA kit as directed by the manufacturer (BD Biosciences, San Diego, CA) [31].

## 3. Results and Discussion

### 3.1. Agroinfiltration, Transient Expression, and Encapsulation of *β*-Glucuronidase (GUS) Protein in a Model Plant and Two Edible Crops

**Figure 1:**
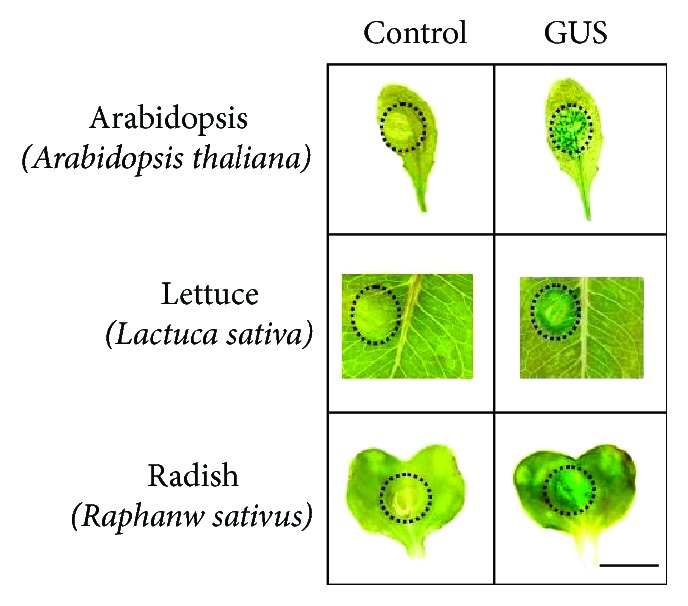
Transient expression of agroinfiltrated GUS in *Arabidopsis*, lettuce, and radish leaves. Transient encapsulation of GUS in leaves of three different plants. In an effort to transiently encapsulate GUS, leaves of *Arabidopsis thaliana*, the first selected to decipher its genome sequences, and two edible crops (lettuce (*Lactuca sativa*) and radish (*Raphanw sativus L.*)) were infiltrated with *A. tumefaciens* (LBA4404 strains, 5 × 10^7^ CFU) transforming a 35S::*GUS* construct (pBI121). Plant leaves infiltrated with nontransformed LBA4404 cells (control, 5 × 10^7^ CFU) served as negative controls. Dotted circles indicate locations of syringe infiltration with *A. tumefaciens.* GUS was detected using a histochemical staining procedure. Blue-stained areas indicate GUS activity. Bar = 6 mm.

Many plants, including *Arabidopsis*, a model plant, are able to express proteins [32] via either stable genetic or transient transformation [33]. *Agrobacterium* has been utilized as a vector to deliver foreign DNA and induce transient expression of recombinant proteins in various plants [34]. In this study, *Arabidopsis* and two edible crops, lettuce and radish, were used as platforms to transiently express GUS and/or antigens. Leaves of these plants were bombarded with *Agrobacterium* harboring a 35S::*GUS* construct via a pressure infiltration. Five days postinfiltration, spatial expression of GUS within the leaves was detected by histochemical GUS staining. Infiltration of radish, lettuce, and *Arabidopsis* leaves with *A. tumefaciens* harboring 35S::GUS constructs resulted in GUS expression in all three plants. Control infiltrations, in which *A. tumefaciens* lacking 35S::GUS constructs was used, did not yield detectable GUS expression (Figure 1). These results confirm the versatility of agroinfiltration for inducing transient expression of transgenes in a variety of plants. Radishes, being edible and easily grown, were used for all following transient expression experiments. As presented in Figure 1, GUS can be agroinfiltrated and transiently expressed in *Arabidopsis*, lettuce, and radish, demonstrating *A. tumefaciens*' broad host range [35]. The Japanese radish (*Raphanus sativus L.*) is an edible leaf vegetable that is grown and consumed throughout the world. Recently, it has been reported that the Japanese radish is the vegetable with the highest per capita consumption within the *Brassicaceae* family. Moreover, it is rich in antioxidant constituents that can potentially prevent several human diseases [36]. Due to its easy growth and edibility, Japanese radish was selected for transient expression of GUS and/or SEB-GUS. Histochemical GUS assays demonstrated that GUS expression is detectable in radish five days after agroinfiltration (Figure 1). Detection of GUS activity using 4-methylumbelliferyl-D-glucuronide (4-MUG) as a substrate indicated that the amount of GUS expression was dramatically elevated to the 0.45 U/mg five days after agroinfiltration [25], which may predict the kinetics or amount of transient protein expression in agroinfiltrated leaves although *in planta* transient transgene expression has not been well quantified [37].

### 3.2. Agroinfiltration of SEB-GUS into Radish Leaves

**Figure 2:**
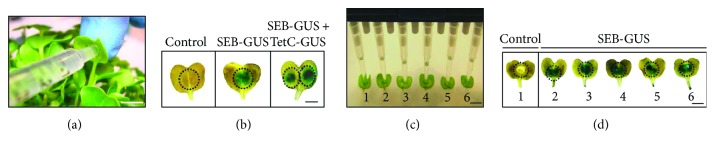
Agroinfiltration of SEB-GUS and TetC-GUS genes into radish leaves. (a) Syringe infiltration of *A. tumefaciens* into radish leaves. Bar = 6 mm. (b) Histochemical staining of radish leaves agroinfiltrated with *A. tumefaciens* (5 × 10^7^ CFU) containing no construct (as a control), 35S::*SEB-GUS* construct, or both 35S::*SEB-GUS* plus 35S::*TetC-GUS* constructs. To simultaneously express two antigens *in planta*, the left half of a single radish leaf was infiltrated with *A. tumefaciens* harboring a 35S::*SEB-GUS* construct while the right half was infiltrated with *A. tumefaciens* harboring a 35S::*TetC-GUS* construct. (c) For higher throughput antigen expression, six isolated radish leaves were concurrently infiltrated with nontransformed *A. tumefaciens* (number 1) as a control and *A. tumefaciens* carrying a 35S::*SEB*-*GUS* construct (numbers 2–6) using a multichannel pipette. Bar = 12 mm. (d) Histochemical staining of leaves simultaneously infiltrated using the multichannel pipette to indicate GUS expression one day after pipetting. Bar = 6 mm.

SEB has been categorized as a biological threat agent in bioterrorism and epidemic outbreaks of food poisoning. Development of a modality that can produce vaccines against SEB in a quick and undemanding way may be an effective strategy to block the SEB spread. In this study, the action of agroinfiltration stamping was displayed by means of pressure infiltration of leaves with an *Agrobacterium*-loaded syringe (Figure 2(a)). Infiltration of radish leaves with *Agrobacterium* containing a 35S::*SEB*-*GUS* construct resulted in recombinant SEB-GUS encapsulation within leaves, as indicated by GUS histochemical staining in the central part of the leaf (Figure 2(b), SEB-GUS). Control leaves agroinfiltrated with *A. tumefaciens* lacking the 35S::*SEB-GUS* construct did not exhibit any staining (Figure 2(b)). Infiltrating each leaflet of a single radish leaf with different *Agrobacterium* transformants, specifically, one containing a 35S::*SEB*-*GUS* construct another containing 35S::*TetC*-*GUS*, allowed a single radish leaf to express two different antigens (SEB and TetC) with distinct spatial encapsulation of the antigens within the leaf (Figure 2(b), SEB-GUS + TetC-GUS), demonstrating the simplicity of using agroinfiltration stamping to create a bivalent vaccine in plants [38]. The throughput of syringe infiltration was increased by using a multichannel pipette to infiltrate six harvested radish leaves in parallel (Figure 2(c)). *A. tumefaciens* either harboring or lacking the 35S::*SEB-GUS* construct was loaded into tips on the multichannel pipette and pressure infiltrated into leaves in a manner similar to that used with syringes. SEB-GUS was detected in the leaves agroinfiltrated with the 35S::*SEB*-*GUS* construct, as indicated by histochemical staining (Figure 2(d)).

In this study, we emphasized the concept of using agroinfiltration stamping to transiently express and encapsulate antigens in radish leaves. The agroinfiltration stamping was illustrated by applying pressure infiltration of *A. tumefaciens* suspension into leaf tissue, accomplished with either a syringe or a multichannel pipette (Figure 2), avoided more complicated techniques like microparticle bombardment [39], which requires gene guns [40] and coating DNA on gold particles. Unlike agroinfiltration stamping, microparticle bombardment will thus make it difficult to simultaneously transfer multiple antigens into a single leaf as well as to bombard antigens in a high-throughput manner. Agroinfiltration is an efficient method for inducing transient expression of multiple antigen transgenes in plant tissue. The concept of high-throughput agroinfiltration system in the study could be applied for producing high level and variety of antigens in the future [41]. Moreover, agroinfiltration can provide milligram amounts of a recombinant protein within a week [42]. This is an important issue because it dramatically accelerates the development of plant lines producing recombinant therapeutics. Importantly, agroinfiltration may even prove suitable for preclinical trials without the need for production of stably transformed plants [14].

### 3.3. Cellular Distribution of SEB-GUS Transient Expression in Radish Leaves

**Figure 3:**
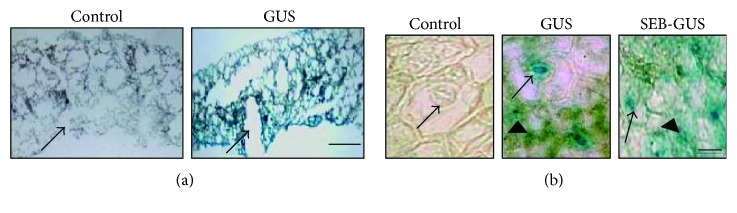
Cellular distributions of transient expression and encapsulation of GUS and SEB-GUS in radish leaves. (a) The majority of GUS-positive cells (blue) were located in the wounded area (arrows) of radish leaves infiltrated with *A. tumefaciens* (5 × 10^7^ CFU) carrying a 35S::*GUS* construct, but not with nontransformed *A. tumefaciens* (control, 5 × 10^7^ CFU). Bar = 0.5 mm. (b) Amplification of wounded areas indicated that GUS or SEB-GUS was detectable in the epidermal (arrowheads) cells, but predominantly expressed in guard cells (arrows) of infiltrated leaves. Bar = 10 *μ*m.

**Figure 4:**
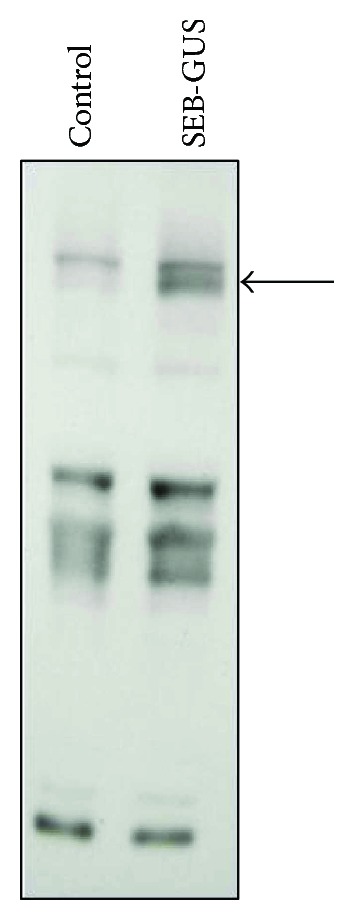
Confirmation of SEB-GUS expression by Western blot analysis. Ground radish leaves (20 *μ*g) infiltrated with *A. tumefaciens* (5 × 10^7^ CFU) carrying a 35S::*GUS* construct (SEB-GUS) or nontransformed *A. tumefaciens* (control, 5 × 10^7^ CFU) were run on a 10% (*w*/*v*) SDS-PAGE and blotted onto a nitrocellulose membrane. The membranes were then probed with mouse monoclonal anti-SEB antibodies. An arrow indicates the molecular weight (96 kDa) of SEB-GUS.

To examine the cellular distributions of GUS and SEB expression, Tissue-Tek Optimal Cutting Temperature- (OCT-) embedded tissue sections of agroinfiltrated radish leaves were stained with 5-bromo-4-chloro-3-indolyl-*β*-D-glucuronic acid cyclohexylammonium salt (X-Gluc). No GUS expression was detected when leaves were infiltrated with nontransformant *A. tumefaciens* (control). GUS expression (indicated by a blue precipitate after X-Gluc treatment) was condensed in the wounded area of radish leaves infiltrated with *A. tumefaciens* carrying a 35S::*GUS* construct (Figure 3(a)). The GUS or SEB-GUS was detectable in epidermal cells, but predominantly expressed in guard cells in the wounded area agroinfiltrated with 35S::*GUS* or 35S::*SEB*-*GUS* constructs, respectively (Figure 3(b)). GUS expression was used as an indicator for SEB expression since constructs were designed to have the SEB coding sequence upstream of the GUS coding sequence in SEB-GUS fusions. Additionally, SEB-GUS expression was detected by a Western blot analysis. Proteins in agroinfiltrated radish leaves were separated using 10% sodium dodecyl sulfate polyacrylamide gel electrophoresis (SDS-PAGE) and reacted with a mouse monoclonal anti-SEB antibody. A band at 96 kDa corresponding to the expression of a SEB- (28 kDa) GUS (68 kDa) fusion protein appeared for leaves infiltrated with *A. tumefaciens* carrying a 35S:*SEB:GUS* construct (Figure 4). Although several protein bands were recognized by a mouse monoclonal anti-SEB antibody, the 96 kDa band is not detected in leaves infiltrated with nontransformant *Agrobacterium* (control). Future work will extract SEB-GUS from infiltrated leaves [43] and conduct Western blot analysis to validate the expression of SEB in leaves. Data from Figures 3 and 4 indicate that SEB-GUS was expressed and encapsulated in radish leaves after agroinfiltration. Through advances in molecular and genetic techniques, protein expression in plants has been optimized for high-level production [44]. Recently, synthesis of codon-optimized bacteria gene in plants is powerful and common [45]. It is conceivable that pathogens and radish sprouts have very different tRNA pools. Thus, synthesis of a codon-optimized gene ought to enhance the production of in plant cells [46]. Moreover, transient expression levels can be elevated by using the cauliflower mosaic virus (CaMV) 35S promoter to drive transgene expression in plants [47]. Previous studies demonstrated the cell type-specific expression of a CaMV 35S-GUS gene in transgenic plants [48]. Here, we showed that epidermal cells and guard cells in CaMV 35S-GUS-transformed radish leaves expressed GUS most readily (Figure 3), which is consistent with GUS expression patterns seen in transgenic tobacco leaves [49].

### 3.4. SEB Immunogenicity and Protective Immunity against IFN-*γ* Production

**Figure 5:**
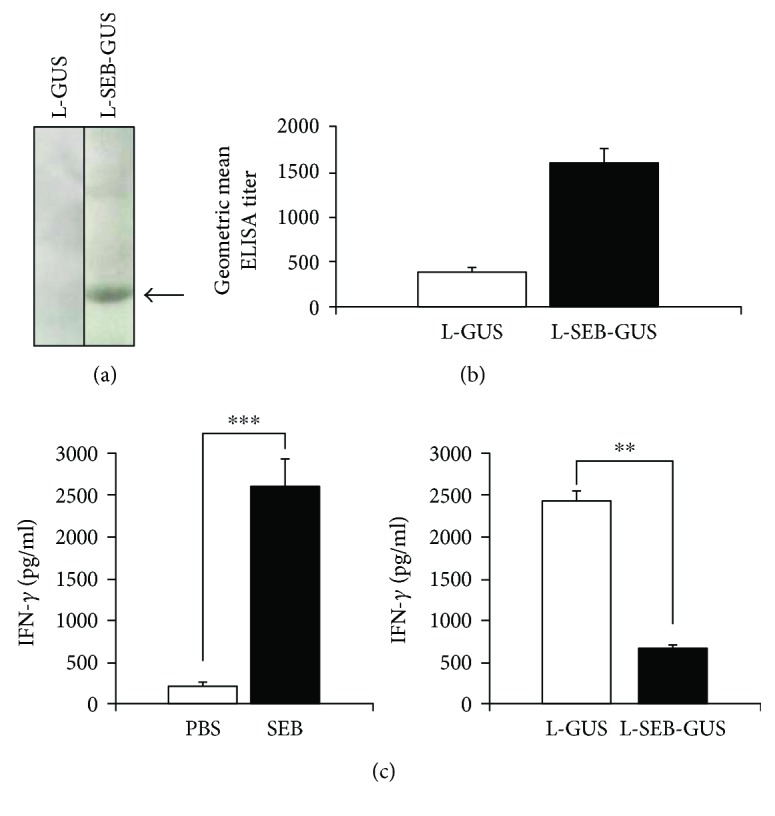
SEB immunogenicity and protective immunity against SEB-induced IFN-*γ* production. (a) Recombinant SEB (15 *μ*g) run on a 10% (*w*/*v*) SDS-PAGE was blotted onto a nitrocellulose membrane and immunoreacted to sera obtained from mice immunized with 25 *μ*l homogenized leaves expressing SEB-GUS (L-SEB-GUS) or GUS (L-GUS) mixed with a CT adjuvant (5 *μ*g/25 *μ*l whole leaves). Recombinant SEB reactive to serum from L-SEB-GUS-immunized mice produces a band (arrow) at approximately 28 kDa, verifying the immunogenicity of SEB. (b) Titers of pooled anti-SEB antibodies from eight immunized mice were qualified by ELISA. The geometric means of ELISA titers were presented. (c) Eight naïve mice were challenged intranasally with 40 *μ*g of recombinant SEB or PBS as a negative control, and the levels of IFN-*γ* in BAL fluids were measured by ELISA. Mice immunized with L-SEB-GUS or L-GUS were challenged intranasally with 40 *μ*g of recombinant SEB overnight. The levels of collected IFN-*γ* in BAL fluids of L-SEB-GUS- and L-GUS-immunized mice were compared by an ELISA assay. Experiments were performed in triplicate. Data were analyzed statistically by Student's *t*-test and presented as mean ± SD (^∗∗^*p* < 0.005; ^∗∗∗^*p* < 0.0005 by Student's *t*-test).

The functionality of SEB-GUS encapsulated in radish leaves as a vaccine was tested. Without purifying SEB from leaves, whole leaves infiltrated with *A. tumefaciens* carrying a 35S::*SEB*-*GUS* (L-SEB-GUS) or a 35S::*GUS* (L-GUS) construct were ground in sterile water, ultraviolet-inactivated, and mixed with cholera toxin, a common adjuvant used for intranasal immunization [50]. The ground leaves were subsequently inoculated into nasal cavities of ICR mice for intranasal immunization. The anti-SEB-GUS antibodies were measurable by a Western blot assay in mouse serum four weeks after intranasal immunization with leaves containing SEB-GUS (Figure 5(a)). Data from enzyme-linked immunosorbent assay (ELISA) indicated that mice immunized with L-SEB-GUS elicited antibody to SEB (Figure 5(b)). No antibodies against SEB were detected in mice immunized with GUS alone. This result demonstrates that SEB expressed in radish leaves can act as a vaccine to confer immunity against SEB. It has been reported that levels of IFN-*γ* in bronchoalveolar lavage (BAL) fluids dramatically increase in mice during SEB-induced inflammation [20]. We intranasally inoculated naïve mice with 40 *μ*g of recombinant SEB or the same volume of phosphate-buffered saline (PBS). The challenge of recombinant SEB significantly augmented the production of IFN-*γ* in BAL fluids (Figure 5(c)). To assess the protective effects of SEB vaccines encapsulated in radish leaves, we next intranasally challenged SEB into mice and measured the change of IFN-*γ* levels in BAL fluids. In mice that had previously been inoculated with leaves containing only GUS, BAL fluid IFN-*γ* levels were 2345.49 ± 64.65 pg/ml after being challenged with SEB. However, in mice that had previously been inoculated with leaves containing SEB-GUS, IFN-*γ* levels in BAL fluid dropped to 586.18 ± 30.69 pg/ml (Figure 5(c)). This result illustrates that SEB vaccine encapsulated in radish leaves confers protection against SEB-induced IFN-*γ* production.

Recently, a number of studies have demonstrated the capability of agroinfiltration to generate recombinant proteins as antigens [51, 52]. These studies focused on increasing recombinant protein yields for purification [53]. Indeed, the antigenicity of proteins relies not only on the protein amounts but also on the protein structures. However, low amounts of protein can provide sufficiently high immunogenicity [54]. In this study, we used homogenized radish leaves expressing SEB, rather than purified recombinant SEB, for immunization. The production of SEB antibodies in immunized mice (Figure 5(a)) demonstrated that agroinfiltration and *in planta* transient expression of SEB is sufficient for leaf tissue to exhibit SEB immunogenicity. Notably, the use of minimally prepared homogenized leaves containing SEB as vaccines can eliminate sophisticated procedures for antigen purification. In fact, agroinfiltration is adding lipopolysaccharide (LPS) from the *Agrobacterium*, which in itself may be a molecule capable of impacting the immune responses [55]. Further works should focus on performing control data for the LPS responses like using SEB from non-LPS sources as a control and comparing its immune response to that from LPS sources. Furthermore, using Western blot and ELISA assays, antibodies against SEB were detectable in mice immunized with homogenized leaves expressing SEB without the addition of an exogenous CT adjuvant (data not shown). This result supports other evidence indicating that leaves contain natural adjuvants such as phyto-saponins [56]. Unfortunately, these immunized mice are unable to suppress SEB-induced IFN-*γ* production (data not shown). Conversely, intranasal immunization of mice with SEB-expressing leaves in conjunction with adjuvant CT not only elicited systemic antibodies to SEB but also offered protective immunity against SEB-induced IFN-*γ* production although it was shown that CT may induce Bell's palsy [57]. Thus, other safe mucosal adjuvants should be analyzed in the future.

GUS has been shown to be an immunogenic protein [58]. In addition, several leaf proteins are antigenic in mice as well [59]. The immunogenicities of GUS and radish proteins in mice immunized with whole leaves containing GUS are undetermined in this study. However, in comparison with immunizations using leaves containing SEB-GUS, mice immunized with leaves containing only GUS elicited high levels of IFN-*γ* after SEB challenge (Figure 5(b)), suggesting that the background of GUS and leaf proteins present in leaves did not inhibit or confound SEB immunogenicity.

## 4. Conclusion

The argoinfiltration stamping was exploited as a novel modality to generate monovalent or bivalent vaccines. Argoinfiltrating gene (SEB and TetC) into radish leaves provides a simple approach for transiently expressing and encapsulating antigens in leaf tissue. This approach avoids the issue of transgene introgression and offers means to generate vaccines in a rapid manner. Moreover, the coexpression of antigens could be applied for analyzing multiple immunological responses to provide new means of vaccine manufacture and delivery without the complicated codelivery procedure following mixing of many expressed antigens. Increased awareness about the prospects of global epidemics and bioterrorism has motivated the development of techniques to create inexpensive vaccines on a rapid, massive scale if necessary [60]. As shown with SEB, transient expression of antigens in plant tissue offers one such method of rapid production. Intranasal immunization with minimally prepared homogenized leaves containing recombinant antigens eliminates the cost and time requirements of antigen purification and avoids the intrinsic problems associated with needle injections. Also, intranasal immunization of mice with ground leaves expressing SEB elicits detectable antibodies to *S. aurues* SEB. However, it had been reported that vaccination via an intranasal route can cause facial nerve paralysis [61]. Therefore, the safety of intranasal administration is worthy to be investigated since the human respiratory tract is not exposed to plant leaves on a routine basis [7]. In addition, the concept of encapsulating proteins/antigens in the leaves instead of purifying them for immunization may benefit vaccine production in the developing countries where cold chain facilities are lacking and emerge as a commercially viable approach for urgent vaccine development.
